# Tobacco control research in Kenya: the existing body of knowledge

**DOI:** 10.11604/pamj.2014.17.155.2707

**Published:** 2014-03-04

**Authors:** Gladwell Koku Gathecha

**Affiliations:** 1 Ministry of Health, Kenya-Division of Non Communicable Diseases, Kenya

**Keywords:** Tobacco control, body of knowledge, Kenya

## Abstract

This review examines the existing tobacco control research done in the country. It further identifies key gaps present in research and gives recommendations on priority research areas required to implement effective tobacco control programmes. Published literature, technical reports and reports by the Ministry of Health were reviewed. It included studies that measure tobacco use and its effects, monitor progress of tobacco control, or articles that are discussing tobacco control policy. The review was conducted in January 2013 and included 18 papers. There are six studies that assessed the prevalence of current tobacco consumption which yielded prevalence's of between 3.8%-19%. Only one study tried to determine an association between Tobacco use and Health. Studies that monitored progress of legislation indicated that the country lacked coordinated efforts for tobacco control, enforcement was weak and monitoring of the existing tobacco legislation was poor. This review has demonstrated that Kenya has made efforts to generate knowledge on tobacco control through research. However there is lack of research that demonstrates the effects of tobacco consumption on health and studies that detail the impact of the various tobacco control interventions.

## Introduction

Tobacco control measures are much more likely to be effective when they are based on solid, locally relevant public health research. A role for research in the immediate future is to develop new knowledge that will assist countries, especially developing countries, in implementing World Health Organization Framework Convention on Tobacco Control (WHO FCTC) provisions and doing so in a cost-effective manner. Research programs that have been designed with the objective of informing policy and tobacco control interventions have demonstrated to be of benefit in the past to the countries that implemented them. Research on disease causation, epidemiology, and educational and policy interventions has contributed significantly to reducing smoking rates in developed countries [1].

Low and middle income counties (LMIC) are yet to achieve their full potential in the use of research for policy development and implementation. Several factors have been noted to be responsible for this including lack of data standardization, weak communications networks, inadequate human and material capacity to conduct research, and lack of funding [2]. As a result, many interventions are implemented without scientific rationale. Additionally, attempts to adopt findings from other developed countries at times results to failure due to the political and social cultural differences.

Tobacco control in Kenya has seen significant gains in the recent past with achievements such as ratification of the WHO FCTC in 2004 and passing of the Tobacco Control Act (TCA) in 2007. The TCA is the legal framework governing tobacco control measures in the country. One of the objectives of the TCA is to promote research and dissemination of information [3]. The country has gone further to develop a national tobacco control action plan which outlines public health policy on tobacco control for Kenya and facilitates implementation of key recommendations of the Tobacco Control Act 2007. A key priority in the act is research, monitoring and evaluation [4].

The Kenya Health Demographic Survey (KDHS, 2008) estimates that eighteen percent of men aged 15 to 45 and less than two percent of women currently take tobacco products. Data from the Global Youth Tobacco Survey conducted in 2007 revealed that among the youth aged 13 to 15 the proportion that were currently using tobacco products was 18.5% [5].

The objective of this review is to provide insight of the body of knowledge on tobacco control available in the country. It is envisaged that this article will identify key gaps present in the area of research and give recommendations to priority research areas required to implement effective tobacco control programmes.

## Methods

To provide this insight, existing published literature, technical reports and reports by the Ministry of Health were reviewed. A search of the internet was conducted using the following search engines Pubmed, the Cochrane Library and advanced. Publications were included if they were original studies and exclude if they were reviews or systematic reviews. Studies from the year 2000 to present were included in the review. An electronic literature search for articles was performed using a combination of subject headings and free text incorporating “Kenya and tobacco”, “Kenya and cigarettes” and “Kenya and smoking”. The search was then extended by manually screening the reference lists and the relevant papers were included.

Study Selection. Two reviewers independently assessed titles, abstracts, and articles for inclusion. I included peer reviewed studies published in English that: measured tobacco use, looked at effects of tobacco use, monitored progress of tobacco control, or articles that were discussing tobacco control policy. This review looks at articles that had a health related aspects and left out articles that looked at tobacco and taxation and alternative livelihoods for tobacco growers.

Among the 42 studies identified in the initial search, nine (21%) publications were manually excluded because the studies were conducted before 2000. Another four were excluded because they were either reviews or systematic reviews. One was excluded because a similar study had been done more recently. After further review, another nine (21%) articles were excluded because they were not related to health resulting in a final sample of 18 papers.

## Current status of knowledge

### Tobacco use

The prevalence of life time tobacco is more than a quarter in three of the studies [6–8] as indicated in Table 1. All studies were conducted in a young population aged between 12 and 32. Only one of the studies had a nationally representative sample [5]. Three of the studies evaluated the use of cigarettes while only one evaluated the use of tobacco products in general [9].

**Table 1:** Prevalence of Lifetime Ever Tobacco use in Kenya

Author, year	Site	Design	Population	Prevalence of Lifetime Ever Tobacco Use	Simple size
Ogwell et al. 2003	Nairobi	Smoking	12-17	31%	1130
Kwamanga et al, 2003	Nairobi	Cigarette	Mean 16.7	32%	5311
MOH-GYTS, 2007	National	Cigarette use	13-15	21.2%	6,768
Otieno et al, 2009	Kisumu	Tobacco products	14-23	18.3	458
Atwoli et al,	Eldoret	Cigarette	18-32	42.8%	500

Prevalence of current tobacco use ranged from 3.8-19% (Table 2). Studies conducted in the rural areas tended to have a lower prevalence than those conducted in the urban regions. Half of the studies were done nationally [5–11]. All the studies were cross-sectional studies. Three of the studies [11–13] did not have a focus on tobacco but were looking at substance abuse in general while the KDHS was examining the health status in general.

**Table 2:** Prevalence of Current Tobacco Consumption

Author, year	Site	Design	Population	Prevalence of Current smoking	Simple size
Kwamanga et al 2003	Nairobi	Cigarette use	Students, Mean 16.7	10.5%	5,311
MOH, GYTS 2007	National	Cigarette use	Students, 13-15	8.2%	6,768
KDHS, 2008	National	Tobacco products	15-49	19%	18,503
Komu et al, 2009	Nairobi	Cigarette use	Students	12.1%	281
Ndetei et al,2010	Rural	Tobacco products	Adolescents	3.8%	800
NACADA, 2012	National	Tobacco products	15--65	9.1%	2,580

Majority of studies done regarding tobacco control research tried to examine the prevalence of tobacco use both current and lifetime. National representative figures are available in three studies that were conducted by government. The Global Youth Tobacco Survey (GYTS) is the most comprehensive study in the country done because it additionally looks at various aspects such as exposure to tobacco advertising, attitudes and perception toward tobacco, cessation among others. Despite the examination of various variables in this study I did not find any attempt at in-depth analysis to determine factors associated with tobacco use. The other two national studies did not have a focus on tobacco. Furthermore, comparison between the three studies is not possible because of the different age groups used for the study.

It is not surprising that majority of the studies have been conducted in the younger population as literature has revealed that smoking is usually initiated in the youth. This together coupled with the ease of conducted school based survey means that such studies are more popular than community based survey [14]. Results from school based surveys also tend to be reliable than household surveys [15]. Such studies build a strong case for the introduction of more vigorous interventions such as introduction of tobacco control in the school curriculum.

Due to differences in methodology it hence follows that there is a varied range of prevalence that does not allow for comparison. The most recent study gives a prevalence of 9.1 but it was carried out in a population aged 15 to 65 hence it is not possible to discern the prevalence among the youth or adult population.

### Health effects of tobacco

The search yielded only one study that was trying to determine an association between Tobacco use and Health [16]. This study found a positive relationship between cigarette smoking and oral leukoplakia. This case control study demonstrated a relative risk (RR) of oral leukoplakia of 9.1 in smokers of filter cigarettes and 9.8 in smokers of non-filter cigarettes. Previous investigations of this study had demonstrated a dose gradient response.

Prominently lacking in the body of research for tobacco control are studies trying to evaluate tobacco related disease incidence and prevalence and mortality. While numerous studies have been done particularly in developed countries in this topic, it is essential to identify the country specific situation so that countries can establish own parameters of the tobacco control problem [1]. Capacity to conduct studies particular analytical epidemiological studies has been identified as a challenge in developing countries [17, 18]. It is envisioned that this will change in the future as epidemiology programmes that offer capacity building and training initiatives will become more prominent in the Country [19].

### Monitoring progress

The Kenya Tobacco Situation Analysis conducted in 2008-2010 demonstrated that the Country lacked coordinated efforts for tobacco control and enforcement and monitoring of the existing tobacco legislation was poor. The finding also revealed that there was substantial industry interference in development and implementation of policy [20]. A qualitative study was undertaken in 2011 and its main objective was to document the progress made by the Government of Kenya in the domestication of the FCTC and the implementation of the TCA 2007. Reports from the study revealed that Kenya had developed multi-sectoral control programmers and strategies but however majority of activities were undertaken by the Ministry of Health with little participation from other Government ministries. Lack of resources and industry interference were cited as barriers to progress [21]. Under the leadership of the WHO a Joint national capacity assessment on the implementation of effective tobacco control policies in Kenya was undertaken in 2011. This assessment looked at status of implementation of tobacco control measures and then evaluated the national capacity to implement provisions of the FCTC. Like previous studies that were monitoring progress, this study also revealed coordination among the players in tobacco control was lacking. Provisions such as establishment of pictorial warnings in cigarette packets had also not been met [22].

There is good attempt at research on monitoring progress of implementation of the FCTC and the TCA. There is consistency in the barriers that hinder effective implementation such as Industry interference and lack of coordination mechanism. Due to the broad nature of the TCA and FCTC, none of the studies gives detail on each specific provision. The studies outlay the current situation of implementation and gives recommendations on ways to ensure implementation is effective. None gives the impact of the FCTC and TCA on tobacco consumption or tobacco related health effects. Nevertheless, this is the ideal time to shift from studies that are monitoring progress to studies that are evaluating the impact of these two measures as the implementation period has been adequate. This is not a unique situation for Kenya as Wilson and colleagues in their review found limited studies that evaluated the impact of tobacco control strategies on tobacco use [23]. Possible explanations for lack of such studies is lack of baseline data before implementation of the two measures that may be as a result of limited funding towards tobacco control before implementation of the FCTC and TCA.

### Tobacco advertising promotion and sponsorship

There are two studies that looked into tobacco, promotion and sponsorship (TAPS) but looked at it from two different angles. The first study looked at sources of and exposure to pro- and anti-tobacco messages in primary schools children [24]. This was a quantitative study done before the enactment of the Tobacco control Act that prohibited TAPS. This study concluded that tobacco advertising was a risk factor for smoking status.

The second study was done over a period of two years and looked at media monitoring and ground monitoring regarding TAPS. Despite the enactment of the TCA it was noted that the Tobacco Industry was still using subtle and indirect techniques to circumvent the existing ban [25].

### Tobacco control and Policy

In 2007, a cross-sectional study was conducted to assess the level of public support for tobacco control policies and to discuss how these findings could be used to influence the legislative process in the passing of tobacco control law in the country [26]. This is a good example of using science to inform policy. Results of this study were used to advocate for passing of the Tobacco Control Bill in parliament as most of the responded favored the bans and restrictions that were to be introduced.

Patel et al. looked at the ability of the tobacco industry particularly British American Tobacco to influence public policy. Legislations on health were found to be delayed and diluted as a result of the high political backing for the Tobacco Industry. This had the overall effect of having limited tobacco control measures that address public health [27]. Research for policy is important especially in tobacco control where the tobacco industry is able to table evidence against tobacco control. Evidence-based policy adoption will continue to be essential to minimizing the burden of tobacco consumption, especially in the world′s poorer countries [28].

## Conclusion

This review has demonstrated that Kenya has made efforts to generate knowledge on tobacco control through research. This has been done through concerted efforts of the government, civil societies, academia and individuals. Of concern however is lack of studies that demonstrate the effects of tobacco consumption. Such information is needed to build for a stronger case for tobacco control as they will be able to demonstrate the effects of tobacco consumption. Additionally, studies that detailed the impact of the tobacco control interventions should be a main focus for the country.
